# Thermal Properties and Dynamic Rheological Characterization of *Dioscorea* Starch Gels

**DOI:** 10.3390/gels10010051

**Published:** 2024-01-10

**Authors:** Bolanle Omolara Otegbayo, Abiola Rebecca Tanimola, Julien Ricci, Olivier Gibert

**Affiliations:** 1 Department of Food Science & Technology, Bowen University, P.M.B. 284, Iwo 232102, Osun State, Nigeria; oladeleabiola12@gmail.com; 2 CIRAD, AGAP Institute, Avenue Agropolis, BP 5035, 34398 Montpellier, France; julien.ricci@cirad.fr (J.R.); olivier.gibert@cirad.fr (O.G.); 3 University of Montpellier, CIRAD-INRAE-Institut Agro, 34398 Montpellier Cedex 5, France; 4 CIRAD, UMR Qualisud, 34398 Montpellier Cedex 5, France; 5 Qualisud, University Montpellier, Avignon Universite, CIRAD, Institut Agro, IRD, Université de La Réunion, 34398 Montpellier Cedex 5, France

**Keywords:** *Dioscorea*, starch gels, dynamic oscillatory, visco-elastic, utilization, gelatinization

## Abstract

Yam (*Dioscorea*. sp.) is an edible starchy tuber with potential for being a commercial source of starch for industrial purposes, but yam starch is underutilized. The dynamic oscillatory and thermal properties of yam starches from sixteen varieties each of *Dioscorea. rotundata*, *Dioscora. alata*, *Dioscorea. bulbifera* and one variety of *Dioscorea. dumetorum* from Nigeria were studied to determine their potential for industrial utilization. The storage modulus, loss modulus, damping factor and complex viscosity as a function of frequency (ω) of the *dioscorea* gels, as well as the onset temperature (*T_o_*), peak gelatinization temperature (*T_p_*), end of gelatinization (*T_C_*), and gelatinization enthalpy of the starches were determined by standard procedures. Results showed that all the *dioscorea* starches showed a typical elastic behavior with the magnitude of G′ greater than G″ while tan δ < 1 in all varieties. Thus, the starch gels were more elastic than viscous. All the starch gels exhibited shear thinning characteristics and showed frequency (ω) independence characteristics of weak gels. *D. rotundata* varieties had the lowest ∆*H_gel_*, while *D. bulbifera* varieties had the highest. The diversity of the visco-elastic and thermal properties of the yam starch gels from different varieties and species can be an advantage in their utilization in both food and non-food industries.

## 1. Introduction

Yams are edible starchy storage tubers that have cultural, economic, and nutritional importance. Starch accounts for 80% (on a dry weight basis) of the yam tuber [1]. Nigeria is the leading producer of yams in the world [2], but yam starch is largely unexploited. This is probably due to the fact that yam starch has not benefitted from the degree of value-added research required to ensure commercial competitiveness on an international scale. This has led to minimal information being available on the functionality and subsequent industrial potential of yam starch. Starch is a very important industrial raw material. They are utilized in diverse industries: as binders, thickeners, viscosity providers, glazing agents, clouding agents, flowing agents, and encapsulating agents in food products; as flocculation and retention aids, sizing agents, coating agents, and adhesives in the paper industry; as printing thickeners and warp sizing agents in the textile industry and as fluid loss control additives in subterranean drilling [3]. The physicochemical and functional properties of starches have been reported to determine their potential uses or applications [4,5]. The rheological and gelatinization properties of starch are important functional properties which determine most of its utilization. The rheological property includes the deformation behavior and flow of foods under well-defined conditions [6]. Components of rheological properties of starches include the pasting characteristics as well as the viscosity and rheological characteristics of starch gel [3]. The rheological characteristics of starch gels are important in product development, quality control, sensory evaluation, and process designing because they enable researchers to identify the molecular structure or distribution of the molecular components of foods and also predict and describe the structural modifications of foods during manufacturing processes [7,8]. They can also quantify both the viscous and the elastic properties of a material at different time scales and frequencies. Thus, rheological characteristics are a valuable tool for understanding the structural and dynamic properties of starch systems without breaking their structural elements [9]. Storage modulus (G′) is a measure of deformation energy stored in the material during the shear process. It represents the elastic behavior of the sample. Loss modulus (G″) is a measure of the deformation energy lost per cycle of sinusoidal deformation; it represents the viscous behavior of the material. The loss tangent (Tan δ) or damping factor (G″/G′) indicates the physical behavior of a system, thus showing the ratio of the viscous and the elastic portion of the viscoelastic deformation [10,11], while complex viscosity (Ƞ*) is the ability of the gel to resist shearing forces. Characterizing the dynamic rheological properties of yam starch can thus give an insight into its potential utilization and the scope of its industrial utilization, thus adding value to yam. The pasting characteristics of yam tubers have been characterized [4,12], and the dynamic oscillatory characteristics of raw yam tubers have also been reported [13]. Thermal properties, starch transition temperature and gelatinization enthalpies usually indicate the order–disorder transition that takes place during the heating of aqueous suspension of starch granules. It identifies the melting, crystallization events and glass transition temperatures of the starch. However, there is a paucity of information on the rheological and thermal properties of yam starch gels from Nigeria. This is necessary in order to explore the industrial potential of yam starch. Hence, this study was focused on determining the dynamic oscillatory and thermal properties of starch gels from sixteen varieties of four yam species *(D. rotundata*, *D. alata*, *D. bulbifera* and one variety of *D. dumetorum*) in order to determine their potential utilization for food and non-food applications. Other physicochemical characteristics of these yam starches such as in situ dynamic oscillatory properties, pasting characteristics, digestibility, granule morphology, and particle size analysis have been reported [13,14]. 

## 2. Results and Discussion

### 2.1. Chemical Composition of Yam Starches

The chemical composition of the yam starches is presented in Table 1. The average mean of crude protein (*D. alata*; 0.07%, *D. bulbifera*; 0.09%, *D. dumetorum*; 0.15%, *D. rotundata;* 0.11%), fat (*D. alata*; 0.13%, *D. bulbifera*; 0.30%, *D. dumetorum*; 0.50%, *D. rotundata;* 0.31%) and ash (*D. alata*; 0.24%, *D. bulbifera*; 0.37%, *D. dumetorum*; 0.19%, *D. rotundata*; 0.36%) of the yam starch samples, respectively, were all less than 1% and were in agreement with the report of previous authors [15,16,17], although variation in these constituents exists among the varieties. The low content of these non-starch constituents reflects the efficiency of the starch extraction process and purity of the starch [18]. The purity of the starch is high when the lipid content is less than 1%, protein content is less than 0.2% and ash is less than 0.5% [18,19]. In terms of the amylose content, there were both intraspecies (within the same species) and interspecies (among the yam species). Among the yam species, there was no significant difference (*p* > 0.05) in the amylose contents of *D. alata, D.bulbifera* and *D. alata* (24.44–26.03%), but the amylose content of *D. dumetorum* was significantly (*p* < 0.05) lower (17.08%). This result agrees with previously reported values for yam starches. Differences in the amylose content of yam starches within the same species may be a result of the genotype, botanical source and activity of the enzymes involved in the biosynthesis of various starches [20,21]. 

### 2.2. Rheological Properties

**Figure 1:**
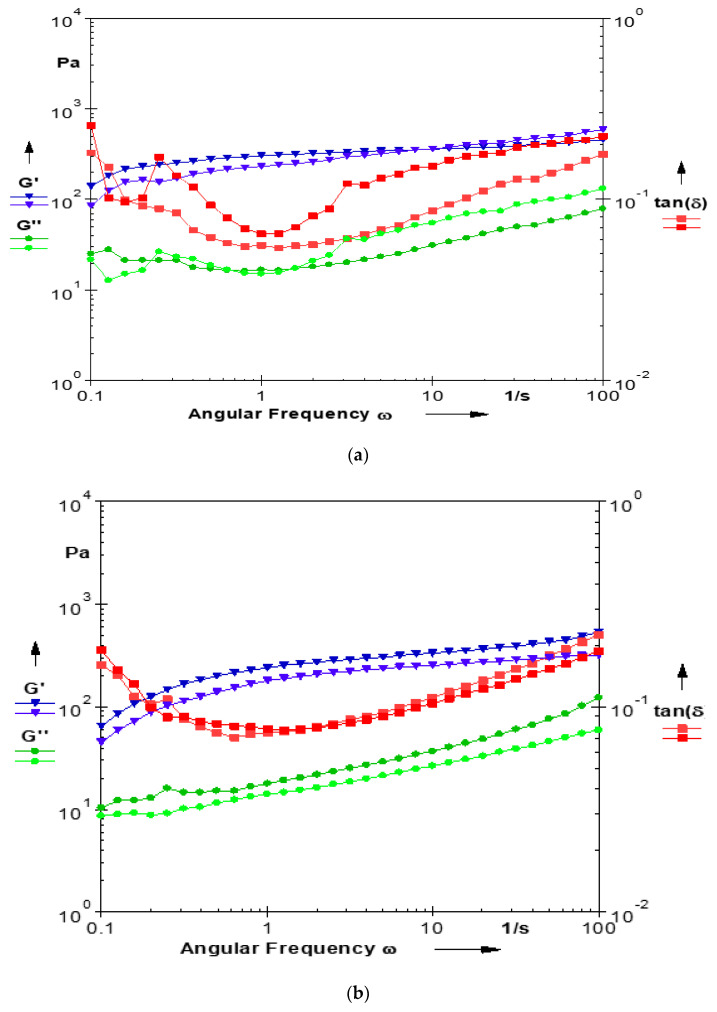

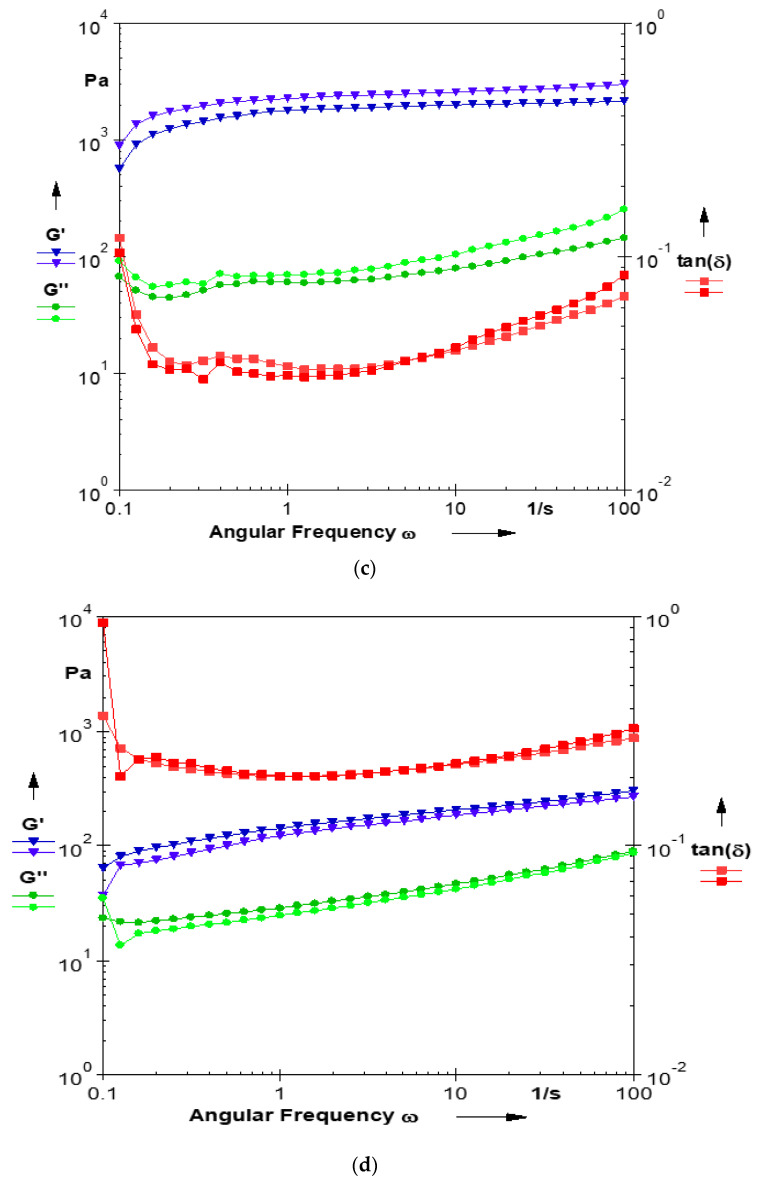
(**a**) Typical mechanical spectra of *D. dumetorum* starch gel (G′—storage modulus (Pa) (duplicate in blue color), G″—loss modulus (Pa)(duplicate in green color), ω—angular frequency (rad/s), tan δ (duplicate in red color). (**b**) Typical mechanical spectra of *D. alata* starch gel (G′—storage modulus (Pa) (duplicate in blue color), G″—loss modulus (Pa) (duplicate in green color), ω—angular frequency (rad/s), tan δ (duplicate in red color). (**c**) Typical mechanical spectra of *D. bulbifera* starch gel (G′—storage modulus (Pa) (duplicate in blue color), G″—loss modulus (Pa) (duplicate in green color), ω—angular frequency (rad/s), tan δ (duplicate in red color). (**d**) Typical mechanical spectra of *D. rotundata* starch gel (G′—storage modulus (Pa) (duplicate in blue color), G″—loss modulus (Pa) (duplicate in green color), ω—angular frequency (rad/s), tan δ (duplicate in blue color).

**Figure 2:**
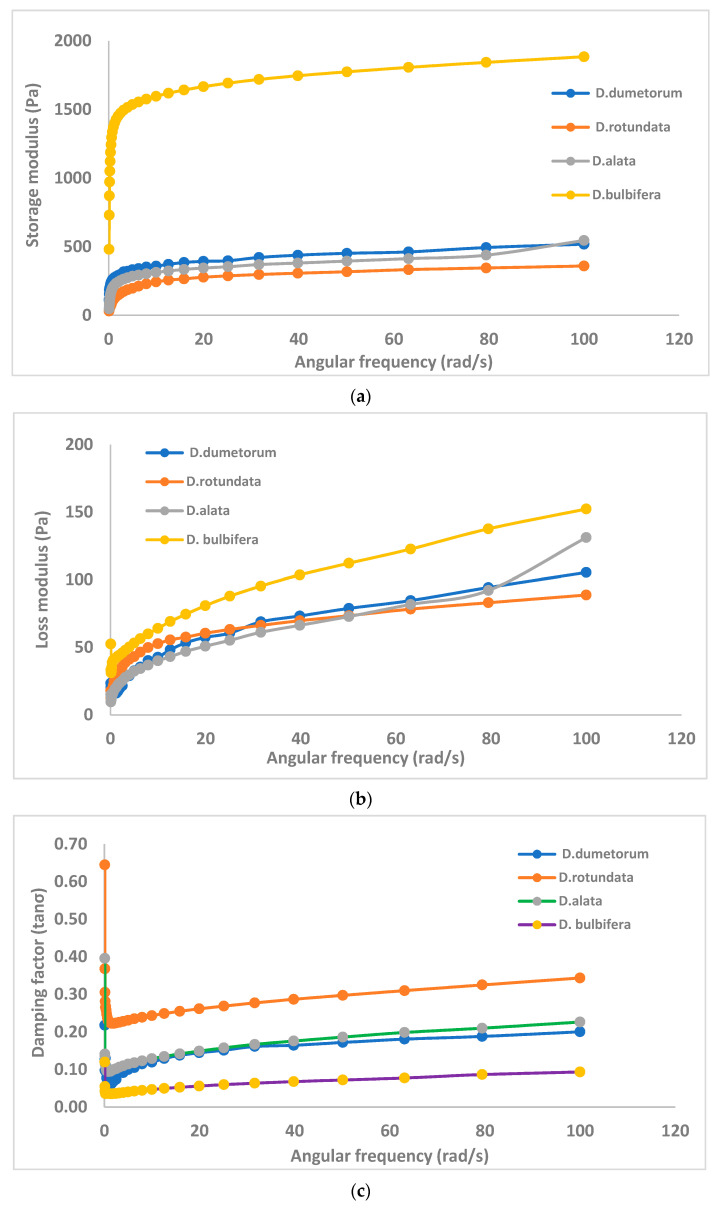

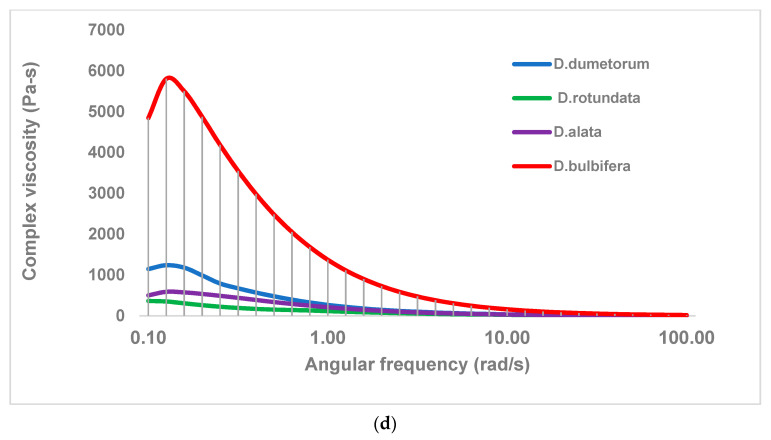
Rheological parameters of yam starches. (**a**) Storage modulus (G′). (**b**) Loss modulus. (**c**) Damping factor (tan δ). (**d**) Complex viscosity (ƞ*).

The rheological properties and the typical mechanical spectra of the yam starch gels are presented in Figure 1a–d and Figure 2a–d. The mechanical spectra showed that the yam starch gels have both elastic and viscous components, but the elastic component dominated with G′ greater than G″, indicating that they are true gels and exhibited visco-elastic properties, which agrees with [22] and also corroborates the report of Moorthy [23] on root and tuber starches. Tangent δ, which is the ratio of the viscous and elastic behavior of the yam starch gels, ranged 0.09–0.34 and was less than unity (<1) in all of the yam starches, indicating that the starch gels were more elastic than viscous [10,11].

**Figure 3:**
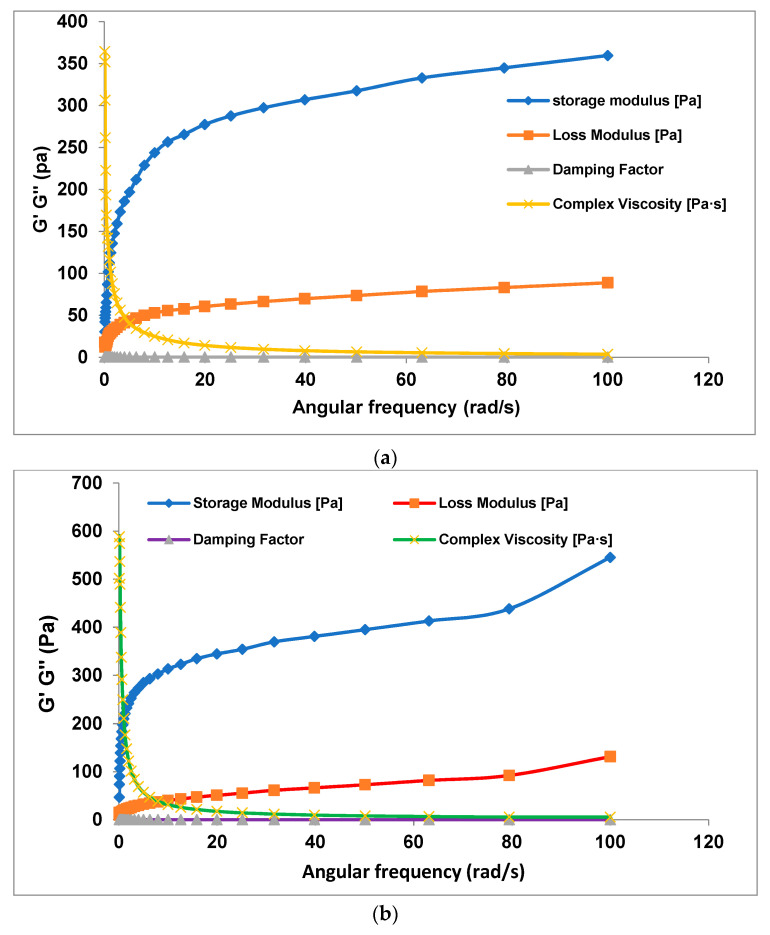

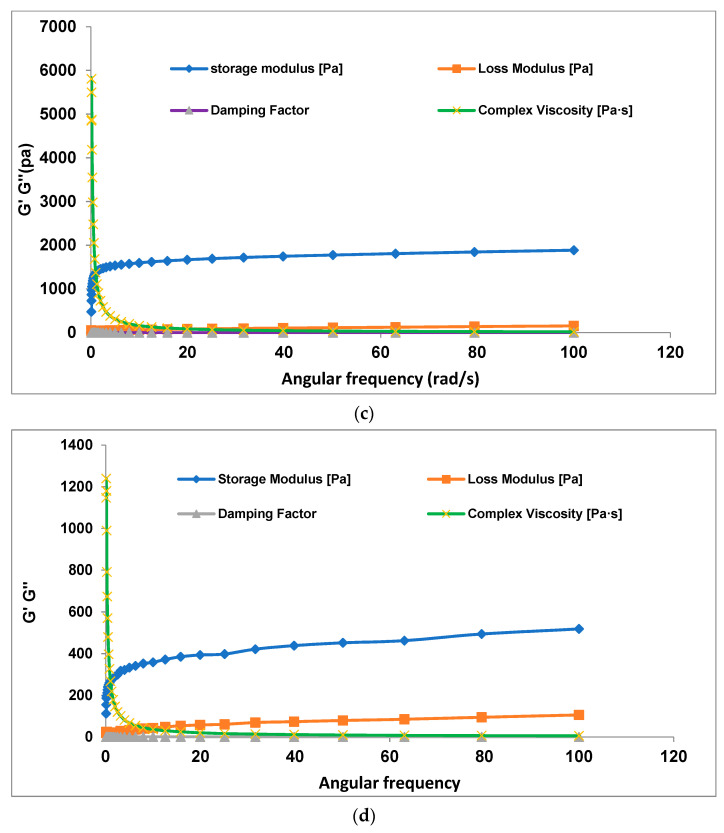
Summary rheological parameters of (**a**) ***D*.** *rotundata* starches, (**b**) *D. alata* starches, (**c**) *D. bulbifera* starches, (**d**) *D. dumetorum* starches.

The G′ and G″ of the starches increased during cooling from 90 to 25 °C, while the lower tan δ of the cooling starches indicates the formation of an elastic gel as a result of starch retrogradation and interactions between leached amylose and starch granules, leading to an increase in gel strength [24,25]. The order of G′ among the yam starch gels was *D. bulbifera* > *D. dumetorum* > D. *alata* > *D. rotundata*, (Figure 2b); thus, *D. rotundata starch gels* had the lowest G′ and therefore were the least elastic among the *dioscorea* starch gels. According to Sundaram et al. [26], the elasticity of a gel quantified as G′ is a measure of the gel’s ability to resist deformation when pressure is applied: the higher the G′, the less it deforms under pressure and the more stored energy it retains. The implication of this in this study is that yam starch gels from varieties in *D. rotundata* species will be more deformable and malleable during processing compared to those from *D. bulbifera* sp. with the highest G′, which can resist deformation during processing. The lower G′ of yam starch gels from *D. rotundata* species may be a result of weaker granule integrity due to greater water uptake and swelling [3]. The higher G′ of yam starch gels from *D. bulbifera* sp. may also be adduced to their large granules [4]. The higher G′ of starch gels from *D. bulbifera* varieties compared with the other yam starch gels implied that they are more elastic than other yam starch gels. The summary of the rheological parameters of each of the yam species is presented in Figure 3a–d.

**Figure 4:**
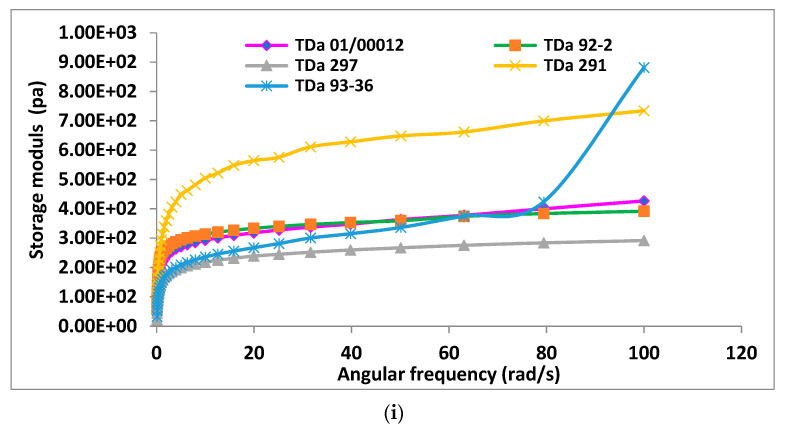

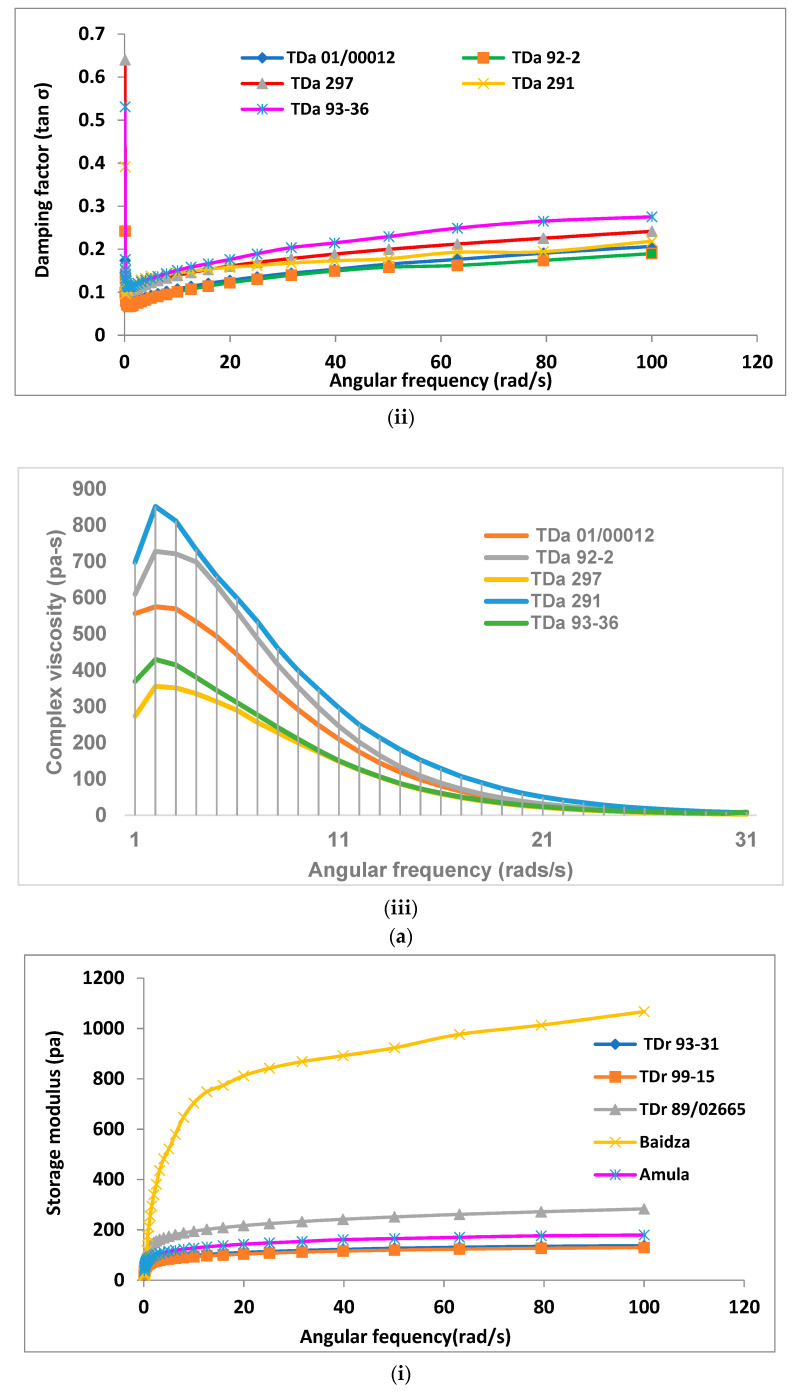

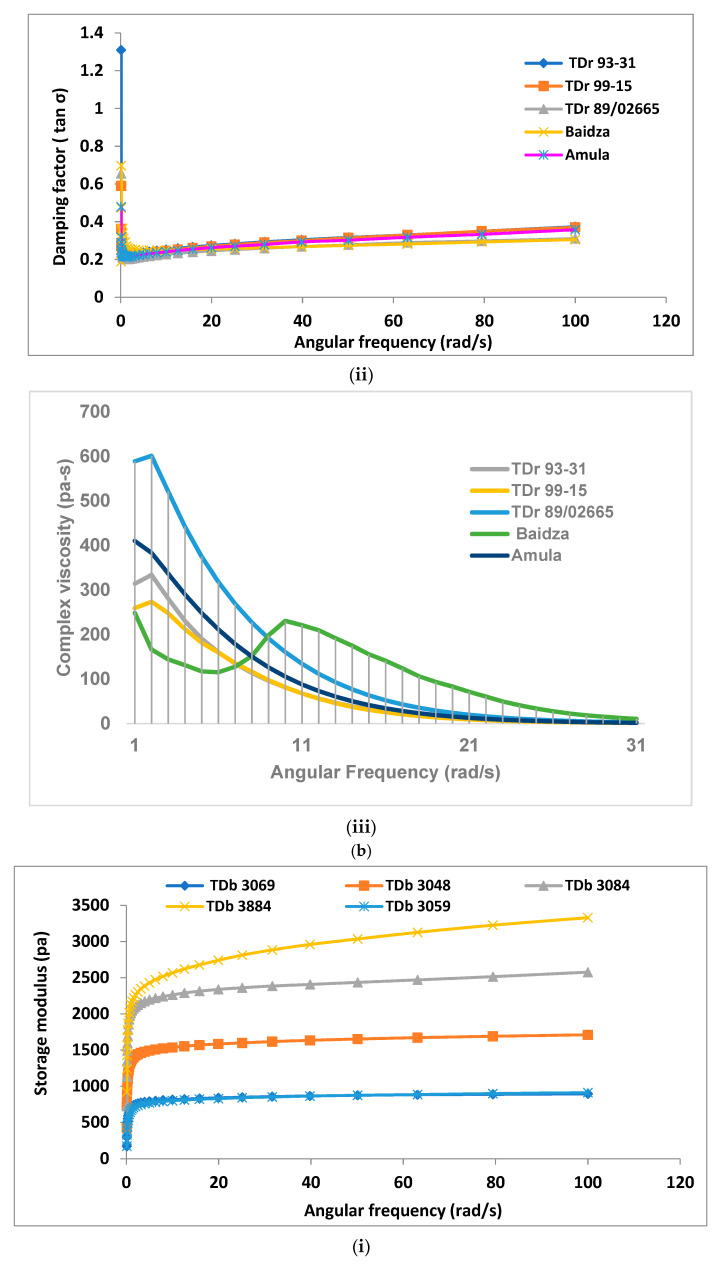

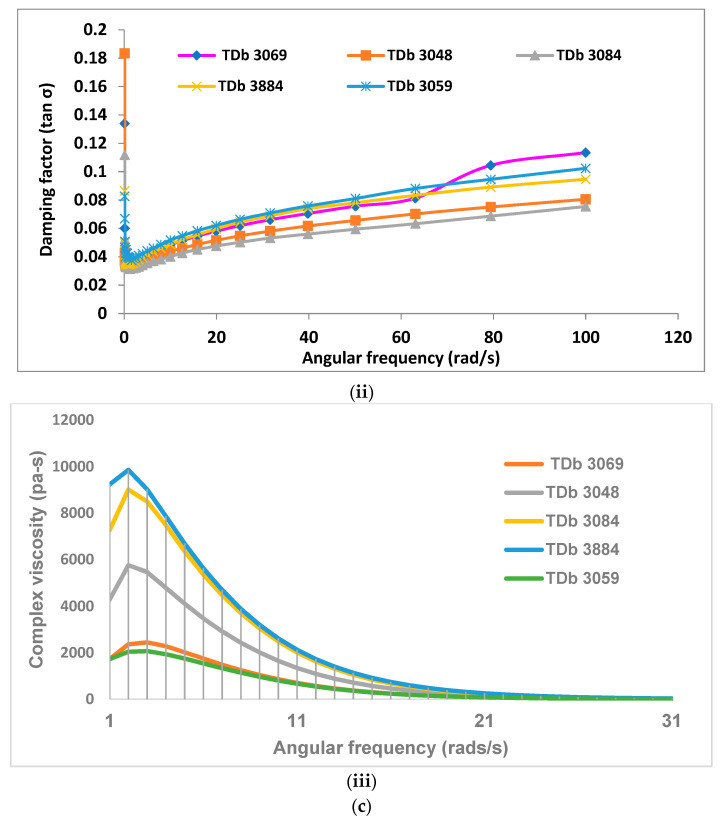
(**a**) Rheological parameters of *D. alata* starches: (**i**) storage modulus (G′), (**ii**) damping factor (tan δ), (**iii**) complex viscosity (ƞ*). (**b**) Rheological parameters of *D. rotundata* starches: (**i**) storage modulus (G′), (**ii**) damping factor (tan δ), (**iii**) complex viscosity (ƞ*). (**c**) Rheological parameters of *D. bulbifera* starches: (**i**) storage modulus (G′), (**ii**) damping factor (tan δ), (**iii**) complex viscosity (ƞ*).

The order of the G′ in the yam starch gels of varieties in *D. bulbifera* was TDb 3884 > TDb 3084 TDb 3048 *>* TDb 3069 > TDb 3059, while the order in *D. alata* was TDa 291 > 92-2 > TDa 01/00012 > TDa 93-36 > TDa 297, and in *D. rotundata*, it was Baidza 89/02665 > Amula > TDr 93-31 > TDr 99-15 (Figure 4a–c).

In terms of the frequency sweep, it was observed that both moduli (G′ and G″) in the yam starch gels increased with increasing frequency but to a lesser extent in *D. bulbifera* starch gels (Figure 4a–c). This is similar to the report of Barual et al. [27]. The increase in the G′ of the starch gels with the increase in frequency showed that they exhibited frequency dependency. The order of the frequency dependence of G′ of the starch gels from the yam species was *D. bulbifera* < *D. dumetorum* < *D. alata* < *D. rotundata*.

Among the *D. alata* varieties, TDa 297and TDa 92-2 were less frequency dependent than TDa 01/00012, TDa 93-36 and TDa 291(Figure 4a), while TDa 291 with the highest G″ was the most viscous of the *D. alata* starch gels.

In the *D. rotundata* sp., TDr 93-31 and TDr 99-15 were less frequency dependent, while Baidza formed the most frequency-dependent starch gel (Figure 4b).

The frequency-dependent characteristics of starch gels have been reported as typical of weak gels [28]. The lower frequency-dependent characteristic exhibited by *D. bulbifera* starch gels was probably a result of the formation of a rigid structure [13], which may be more resistant to deformation due to the stress increase or mechanical fragmentation or shear during processing. Previous authors [8,29] reported that the frequency dependence of a starch can provide important information on the structure of the gel.

Starch gels which are frequency independent indicate a solid-like gel and a true gel, while frequency-dependent gels may imply a gel structure with molecular entanglements. Hence, they act as solid at higher frequencies (low tan δ) and as a liquid at lower frequencies (high tan δ) [30]. According to Keetels et al. [31], the stronger the dependence of G′ on frequency, the lower the stiffness of the gel. The implication of this in this study is that that the starch gels of yam varieties from *D. bulbifera, D. dumetorum* and *D. alata* were stiffer or stronger compared with that of *D. rotundata* with starch gels from varieties of *D. bulbifera* being the strongest and stiffest.

Tan δ was less than unity (<1) in all of the yam starch gels; this implied that the starch gels were more elastic than viscous and formed a typical gel network. The higher the tan δ, the more viscous the starch gel. Chakraborty et al. [32] reported that a lower tan δ indicates a typical gel network and starch gels with stronger gelling ability. Of all the starch gels from the yam species studied, starch gels from varieties of *D. rotundata* had the highest tan δ indicating the most viscous starch gels, while *D. bulbifera* varieties had the lowest; hence, it produced the most elastic gel (Figure 2b). The tan δ value of *D. bulbifera* (0.09–0.11) implied that the yam starch gels can be classified as glassy and crystalline polymers which are usually strong and rigid gels, while those of *D. bulbifera, D. dumetorum* and *D. alata* with tan δ (0.1–0.3) can be classified as amorphous polymers which form weak gels [33].

Among the *D. alata* varieties, the order of tan δ was TDa 93-36 > TDa 297 > TDa 291 > TDa 92-2. This implies that TDa 93-36 was the most viscous, while TDa 92-2 formed the most elastic gel (Figure 4a). Amongst the *D. rotundata* varieties, TDr 99-15 and Amula had the highest tan δ and hence were the most viscous, while tan δ of TDr 93-31, Baidza and TDr 89/02665 (Figure 4b) were lower; hence, they formed more elastic gels. Amongst the *D. bulbifera* starches, the order was 3069 > 3059 > 3884 > 3048 > 3084 (Figure 4c). *D. bulbifera* starch gels had the lowest tan δ among all the yam starches. 

From an industrial application point of view, the result of the tan δ of these yam starch gels implies that the starch of *D. bulbifera* (with lower tan δ) will be very elastic, not easily deformable or stiff and may be difficult to process, while *D. rotundata* starch gels (with higher tan δ) will be less elastic, more deformable and will be easier to process than the other starch gels. This will also influence their utilization as thickeners, binders or fillers in the food and pharmaceutical, industries or other non-food industries such as cosmetic or textile industries or for other commercial uses.

In terms of the Ƞ* which is the ability of the gel to resist shearing forces (it also reveals soft–solid structure rigidity differences between starch gels). The complex viscosity of all the starches decreased as the frequency increased. This indicates that all the *yam* starches underwent shear thinning to viscosity. A viscosity decrease (thin out) during an increase in shear force is called shear thinning [34]. Among the yam starch gels, the complex viscosity was in the order *D. bulbifera > D. alata > D. dumetorum > D. rotundata*. This showed that *D. bulbifera* starch gels had the highest complex viscosity, implying that it had the highest resistance to shear thinning and exhibited the strongest pseudoplastic behavior. There was intraspecies variation in the complex viscosity of the starch gels, but generally, all the varieties within *D. bulbifera* species had higher complex viscosity compared to starch gels from the other yam species; hence, they had higher resistance to shear thinning than all varieties within the other yam species. The implication of this is that since the rheological properties were evaluated under conditions that were very close to industrial processing conditions, it means that all the yam starch gels except *D. bulbifera* starches will have little resistance to shear thinning or mechanical fragmentation (breakdown in viscosity) during mechanical shear or during processing.

Differences in the viscoelastic properties of the starch gels or different responses to dynamic moduli may be attributed to their starch granule properties (rigidity and integrity), intergranular interaction such as entanglement between surface molecules of adjacent granules in gelatinized starches and biological origin of the starches and amylose contents [13,34,35]. 

### 2.3. Thermal Properties of Dioscorea Starches

DSC is usually used to study the thermal behavior of starches. The thermal properties of starches usually indicate the order–disorder transition that takes place during the heating of an aqueous suspension of starch granules. It identifies the melting, crystallization events and glass transition temperatures of the starch. The transition temperatures (*T_o_*, *T_p_*, *T_c_*) are related to the internal crystalline structure of the starch and its thermal stability [36]. The enthalpy of gelatinization (∆*H_gel_l*) gives an overall measure of crystallinity (quality and quantity of crystallites), and it is an indicator of the loss of double helical order; that is, it is a loss of molecular order within the granule or the energy needed for the dissociation of the double helical order. The peak gelatinization temperature *T_p_* is an indication of the crystallite quality (double helix length). The onset temperature (*T_o_*) range was 71.63–76.80 °C, the peak gelatinization temperature (*T_p_*) range was 76.32.2–81.40 °C, the end of gelatinization (*T_C_*) range was 80.36–85.86 °C (Table 2), the gelatinization enthalpy or total energy ∆*H_gel_* range was 16.33–17.67 J/g, while the gelatinization temperature range *R* was 7.82–9.07 °C. The transition temperatures *T_o_*, *T_p_*, *T_C_* and ∆*H_gel_l* of these yam starches agreed with [37] but the ∆*H_gel_* was less than the 22–25 J/g reported for *D. trifida* [38] and also lower than the range of 23.08–31.23 J/g [39] reported for some Chinese yam starches. In addition, higher transition temperatures (*T_o_*, *T_p_*, *Tc* and R (*Tc* − *T_o_*)) than those reported in this study were reported for nine yam starches from *D. opposita* species [40]. On average, in this study, yam varieties from *D. alata* (TDa) and *D. bulbifera* (TDb) had higher *T_o_* and *T_p_* than *D. rotundata* (TDr), while TDr had a higher enthalpy of gelatinization *(*∆*H_gel_l*) than the other yam starches. The higher transition temperature of starches from TDa and TDb could be attributed to their less crystallinity (due to higher amylose, as seen in Table 2) and could also be the result of a more rigid granular structure [13]; hence, they did not gelatinize easily. The high *T_p_* values of these starches may also suggest that they have longer chain lengths, since starches with longer chains have been reported to require higher temperature to dissociate completely compared with that required for shorter double helices [41]. The lower transition temperature and higher ∆*H_gel_* of *D. rotundata* could also be attributed to its higher crystallinity due to higher amylopectin. The industrial implication of its lower ∆*H_gel_* is that it will have lower energy requirements for starch gelatinization during processing. High Δ*H*_gel_ values signify the energy required for disentanglement and melting of the double helical structures formed during gelatinization [42]. In the case of D*. dumetorum* (TDd), the high ∆*H_gel_* may be attributed to its low amylose (Table 1) and higher amylopectin content (since low amylose indicate higher amylopectin). This is because starches with lower amylose content have more crystalline points; this high degree of crystallinity provides the structural stability which makes the granule more resistant to gelatinization. Hence, more energy is needed to initiate melting in the absence of amylose-rich amorphous regions, requiring a higher gelatinization temperature [43,44]. There was no significant difference (*p* > 0.05) among the yam starches in terms of the *R* (*T_c_ − T_o_*) value, although TDr starches had the highest. Differences in the *R* values of starches from different species/varieties may imply a higher degree of heterogeneity (crystallites with different sizes and strength) and stability among the crystallites [39,45,46,47]. Therefore, the gelatinization temperature range of yam starches with large *R* values indicates that they have a higher degree of heterogeneity among their crystallites. Intraspecies variations in the thermal properties of these *dioscorea* starches were observed. For instance, among the *D. alata* species, TDa 92-2, TDa 291, and TDa 01/00012 had higher transition temperatures and ∆*H_gel_* compared to other varieties. The variation in transition temperatures and ∆*H* J/g among varieties of the same cultivars has been adduced to reasons such as the interaction between starch and non-starch components, the molecular architecture of the crystalline region, the amylose contents, the length of amylopectin chains, and the shape and size of granules [25,48,49]. The variation in the thermal properties of these yam starches showed that they will have different energy requirements as raw materials during processing (such as sterilization, pasteurization, cooking time), heat conductivity requirements, cost reduction, food quality and safety, which can influence their selection for specific applications.

## 3. Conclusions

Rheological analyses of the yam starch gels from the sixteen yam varieties revealed that all the yam starches showed a typical elastic behavior with G′ > G″, while tan δ was less than unity (<1) in all of them, indicating that the starch gels were more elastic than viscous. However, there were differences in the viscoelastic properties of the gels or different responses to dynamic moduli within varieties in a species (intraspecies variation) and among the species (interspecies variation). The different responses of the yam starch gels to dynamic moduli may be a result of their botanical origin and starch granule properties (rigidity and integrity). The complex viscosity (Ƞ*) of all the starch gels decreased, implying shear thinning characteristics. *D. bulbifera* varieties had the highest complex viscosity values, which implied that these yam starches will exhibit the least shear thinning properties during processing; hence, they may be useful for products requiring high viscosity. Varieties within the *D. bulbifera* species exhibited the least frequency-dependent characteristics, while the G′ of *D. rotundata*, *D. dumetorum* and *D. alata* starch gels increased with the increase in frequency (ω), implying there being more frequency-dependent characteristics of weak gels. Due to the lower tan δ of *D. bulbifera* starch gels, they will be more elastic and stiffer and may be difficult to process when compared with starch gels from *D. rotundata*, *D. alata* and *D. dumetorum* with higher tan δ, which may be more malleable and amenable during processing. Thus, starch gels from *D. rotundata* and *D. alata* varieties may be used in food industries as thickeners binders, gelling, glazing, clouding agents, etc., while *D. bulbifera* starches may be utilized as viscosity builders and could also be used in textile industries as stiffeners. Diversity in the viscoelastic properties of the yam starch gels from different varieties and species can be an advantage in their utilization in both food and non-food industries. In terms of the thermal properties of the yam starch gels, the higher transition temperatures of the starch gels of *D. bulbifera*, *D. dumetorum* and *D. alata* compared to that of *D. rotundata* implied that they will have more energy requirement to affect the gelatinization of their starches during processing. The variation in the thermal and rheological properties of these yam starch gels implies that yam starch from the different varieties of these yam species can be used as raw materials for specific applications in the food industry and non-food industry.

## 4. Materials and Methods

### 4.1. Materials

Sixteen varieties each of *D. rotundata* (TDr 93-31,TDr 99-15, TDr 89/02665, Amula, Baidza), *D. alata* (TDa 291, TDa 297, TDa93-36, TDa 01/00012), *D. bulbifera* (TDb 3059, TDb 3069, TDb 3048, TDb 3884TDb 3084), and one variety of *D. dumetorum* (TDd) (esuru funfun) were collected from the IITA yam germplasm of International Institute of Tropical Agriculture (IITA), Ibadan, Nigeria.

### 4.2. Methods

#### 4.2.1. Yam Starch Extraction

Starch was extracted from the sixteen varieties of yam, respectively, as described in Otegbayo et al. [50]. The yam tubers were diced into small cubes and homogenized with distilled water (excess) in a blender. The blender was used in short bursts to avoid heating the starch. The slurry was filtered through a muslin cloth; then, the slurry was poured in containers and allowed to sediment for about 2 h. The supernatant was discarded; then, the sediment which is the yam starch was washed several times with distilled water to remove impurities from the starch. The starch was oven dried at a temperature of 30 °C for 48 h. The dried starch samples were pulverized with the Waring blender and ground to pass through a 100 µm mesh size sieve. The milled starch samples were packed in a zip-lock bag and kept in a desiccator prior to analyses.

#### 4.2.2. Proximate Composition of Starch Samples

The proximate composition (moisture, protein, ash, fat) of the starch samples was determined in triplicate by AOAC [51] methods.

#### 4.2.3. Amylose Content

The amylose content of the yam starch samples was determined as described by Mestres [38,52] using a differential scanning calorimetry (DSC, Perkin-Elmer DSC 7, Norwalk, CT, USA). First, 10–11 mg of starch, 50 μL of lyso-phospholipid and 2% distilled water (*w*/*v*) was loaded into stainless steel sealed pans. A pure amylose potato starch sample (Avebe, Veendam, The Netherlands) was used as a standard. The samples were hermetically sealed and allowed to stand for 1 hour before heating in the DSC. The DSC analyzer was calibrated using indium, and an empty aluminum pan was used as reference. The sample pan and the empty reference pan were heated from 25 to 160 °C at a scanning rate of 10 °C per min and held for 2 min at 160 °C and cooled to 60 °C at 10 °C min^−1^. The amylose/lyso-phospholipid complex enthalpy variation (Δ*H*) for samples and standard were determined. The amylose content was computed as the percentage ratio of the Δ*H* amylose of the sample to the Δ*H* amylose of the standard. The analysis was performed in duplicate, and mean values were calculated.

#### 4.2.4. Thermal Properties

The thermal properties of the starch samples were investigated by differential scanning calorimetry (DSC, Perkin-Elmer DSC 7, Norwalk, CT, USA). First, 10–11 mg of starch and 50 μL of 2% (*w*/*v*) lyso-phospholipid was loaded into stainless steel sealed pans. The samples were hermetically sealed and allowed to stand for 1 hour before heating in the DSC. The DSC analyzer was calibrated using indium, and an empty aluminum pan was used as reference. The sample pan and the empty reference pan were heated from 25 to 160 °C at a scanning rate of 10 °C min^−1^, held for 2 min at 160 °C and cooled to 60 °C at 10 °C min^−1^. The gelatinization enthalpy variation (Δ*H*) and the onset gelatinization temperature (To) of each sample were determined on each thermogram within the 55–90 °C range of the linear baseline. The analysis was performed in duplicate, and mean values were calculated.

#### 4.2.5. Preparation of Starch Gels

Starch paste (8% db) was prepared by dispersing the starch in 20 ml of distilled water and gelatinized for 15 min in a water bath maintained at 95 °C with continuous stirring.

#### 4.2.6. Rheological Characterization of Starch Gels

The pastes were immediately transferred to the rheometer (Physica MCR 301 rheometer). The rheometer was equipped with a cone plate geometry (diameter = 35 mm, cone angle = 1°, measurement gap = 1 mm) and was computer controlled with Rheowin195 3 job manager software. The starch was cooled from 95 to 25 °C on the rheometer, and the dynamic moduli (G′ and G′’) was determined. A frequency sweep was carried out from (0.1 to 100 Hz). The viscoelastic range for each yam variety was checked by normal force sweep (0.5 to 100 N) and strain sweep (0.01 to 100%). 

#### 4.2.7. Statistical Analysis

Data generated were subjected to analysis of variance using SAS package version 9.1 (Statistical Analysis Systems of SAS Institute, Inc., Cary, NC, USA). Analysis of variance and means separations were calculated by the general linear model (GLM) procedure.

## Figures and Tables

**Table 1:** Chemical composition of yam starches.

* Species/Varieties	** Amylose	Moisture Content	% Crude Protein	% Crude Fat	% Total Ash
*D. dumetorum*	17.08 ± 0.24	9.14 ± 0.35 ^b^	0.15 ± 0.00 ^a^	0.50 ± 0.00 ^a^	0.19 ± 0.01 ^b^
*D. alata*					
TDa 92-2	22.53 ± 0.01 ^b^	14.06 ± 0.01 ^b^	0.07 ± 0.00 ^b^	0.00 ± 0.00 ^c^	0.17 ± 0.00 ^d^
TDa 01/00012	26.02 ± 1.40 ^a^	13.37 ± 0.03 ^d^	0.07 ± 0.00 ^b^	0.30 ± 0.02 ^a^	0.30 ± 0.01 ^a^
TDa 297	23.60 ± 0.28 ^ab^	14.17 ± 0.03 ^a^	0.08 ± 0.00 ^a^	0.00 ± 0.00 ^c^	0.23 ± 0.00 ^c^
TDa 291	24.75 ± 0.40 ^ab^	12.34 ± 0.04 ^e^	0.07 ± 0.00 ^b^	0.00 ± 0.00 ^c^	0.24 ± 0.00 ^b^
TDa 93-36	25.31 ± 1.58 ^a^	13.58 ± 0.03 ^c^	0.07 ± 0.01 ^b^	0.15 ± 0.02 ^b^	0.17 ± 0.00 ^d^
Mean	24.44 ± 1.49 ^a^	13.50 ± 0.73 ^a^	0.07 ± 0.00 ^b^	0.09 ± 0.13 ^b^	0.22 ± 0.05 ^ab^
*D. bulbifera*					
TDb 3069	12.02 ± 0.01 ^d^	0.07 ± 0.00 ^c^	0.00 ± 0.00 ^c^	0.25 ± 0.00 ^d^	12.02 ± 0.01 ^d^
TDb 3084	12.42 ± 0.02 ^c^	0.08 ± 0.00 ^b^	0.00 ± 0.00 ^c^	0.31 ± 0.00 ^a^	12.42 ± 0.02 ^c^
TDb 3048	13.72 ± 0.01 ^a^	0.09 ± 0.01 ^a^	0.07 ± 0.00 ^b^	0.26 ± 0.00 ^c^	13.72 ± 0.01 ^a^
TDb 3884	13.63 ± 0.03 ^b^	0.06 ± 0.00 ^d^	0.55 ± 0.01 ^a^	0.25 ± 0.00 ^d^	13.63 ± 0.03 ^b^
TDb 3059	11.98 ± 0.01 ^e^	0.06 ± 0.00 ^d^	0.00 ± 0.00 ^c^	0.30 ± 0.00 ^b^	11.98 ± 0.01 ^e^
Mean	12.75 ± 0.86 ^a^	0.07 ± 0.01 ^b^	0.12 ± 0.24 ^b^	0.27 ± 0.03 ^a^	12.75 ± 0.86 ^a^
*D. rotundata*					
TDr 99-15	10.87 ± 0.03 ^e^	0.08 ± 0.00 ^b^	0.03 ± 0.01 ^a^	0.33 ± 0.00 ^a^	10.87 ± 0.03 ^e^
TDr 89/02665	16.58 ± 0.02 ^a^	0.07 ± 0.00 ^c^	0.02 ± 0.00 ^b^	0.32 ± 0.00 ^b^	16.58 ± 0.02 ^a^
TDr 93-31	13.74 ± 0.02 ^d^	0.06 ± 0.01 ^d^	0.00 ± 0.00 ^c^	0.21 ± 0.01 ^e^	13.74 ± 0.02 ^d^
Baidza	15.00 ± 0.04 ^c^	0.06 ± 0.01 ^d^	0.00 ± 0.00 ^c^	0.27 ± 0.00 ^c^	15.00 ± 0.04 ^c^
TDr Amula	16.20 ± 0.03 ^b^	0.09 ± 0.00 ^a^	0.00 ± 0.00 ^c^	0.26 ± 0.00 ^d^	16.20 ± 0.03 ^b^
Mean	14.48 ± 2.30 ^a^	0.07 ± 0.01 ^b^	0.01 ± 0.01 ^b^	0.28 ± 0.05 ^a^	14.48 ± 2.30 ^a^

* Mean (±standard deviation) of triplicate analysis. ** Means with same letters in the same column are not significantly different (*p* > 0.05) at 5% level of significance. TDr—Tropical *dioscorea rotundata*, TDa—Tropical d*ioscorea* a*lata*, TDb—Tropical *dioscorea bulbifera*, TDd—Tropical *dioscorea dumetorum*.

**Table 2:** Thermal properties of yam starches.

Species (Varieties)	* *T_o_* (°C)	*T_p_* (°C)	*T_c_* (°C)	*R* (*T_c_* − *T_o_*) °C	Δ*H* (J/g)
*D. dumetorum*	76.80 ± 0.19	81.400 ± 0.81	85.86 ± 0.33	9.07 ± 0.13	17.41 ± 0.77
*D. alata*					
TDa 92-2	76.64 ± 0.45 ^a^	79.84 ± 0.23 ^a^	83.33 ± 0.35 ^a^	6.69 ± 0.10 ^b^	17.67 ± 2.67 ^a^
TDa 01/00012	75.40 ± 0.12 ^b^	78.42 ± 0.12 ^b^	81.56 ± 0.16 ^b^	6.16 ± 0.04 ^b^	18.71 ± 0.53 ^a^
TDa 297	70.92 ± 0.21 ^d^	79.57 ± 0.37 ^a^	79.64 ± 0.45 ^c^	8.72 ± 0.25 ^a^	15.69 ± 0.35 ^a^
TDa 291	73.39 ± 0.26 ^c^	78.00 ± 0.24 ^b^	82.82 ± 0.50 ^a^	9.43 ± 0.24 ^a^	16.36 ± 0.69 ^a^
TDa 93-36	71.97 ± 0.74 ^d^	76.75 ± 0.11 ^c^	81.79 ± 0.25 ^b^	9.82 ± 0.98 ^a^	17.31 ± 1.63 ^a^
** Mean	73.66 ± 2.24	78.51 ± 1.19	81.83 ± 1.37	8.17 ± 1.59	17.15 ±
*D. bulbifera*					
TDb 3069	73.89 ± 0.01 ^a^	79.17 ± 0.00 ^b^	84.24 ± 0.24 ^a^	10.35 ± 0.23 ^a^	16.23 ± 0.34 ^a^
TDb 3084	73.90 ± 0.52 ^a^	79.42 ± 0.35 ^ab^	83.82 ± 0.53 ^a^	9.92 ± 0.01 ^a^	16.87 ± 1.42 ^a^
TDb 3048	76.66 ± 3.46 ^a^	79.09 ± 0.12 ^b^	82.60 ± 0.16 ^b^	5.94 ± 3.36 ^a^	17.58 ± 0.19 ^a^
TDb 3884	77.60 ± 3.18 ^a^	79.92 ± 0.12 ^a^	83.77 ± 0.14 ^a^	6.17 ± 3.32 ^a^	16.19 ± 0.68 ^a^
TDb 3059	76.93 ± 3.90 ^a^	79.50 ± 0.24 ^ab^	83.64 ± 0.22 ^a^	6.71 ± 3.68 ^a^	14.76 ± 2.13 ^a^
Mean	75.80 ± 2.64	79.42 ± 0.34	83.61 ± 0.61	7.82 ± 2.88	16.33 ± 1.32
*D. rotundata*					
TDr 99-15	72.97 ± 0.04 ^a^	77.00 ± 0.47 ^a^	82.26 ± 0.10 ^a^	9.30 ± 0.06 ^a^	17.47 ± 0.76 ^a^
TDr 89/02665	70.82 ± 0.02 ^d^	75.42 ± 0.12 ^a^	80.07 ± 0.08 ^c^	9.25 ± 0.06 ^a^	17.66 ± 0.23 ^a^
TDr 93-31	71.91 ± 0.11 ^b^	76.25 ± 0.11 ^a^	80.99 ± 0.12 ^b^	9.08 ± 0.23 ^a^	18.40 ± 0.06 ^a^
Baidza	71.46 ± 0.32 ^c^	77.42 ± 3.18 ^a^	78.95 ± 0.49 ^d^	7.50 ± 0.18 ^c^	17.68 ± 0.84 ^a^
TDr Amula	71.37 ± 0.01 ^c^	75.50 ± 0.24 ^a^	79.54 ± 0.21 ^cd^	8.18 ± 0.22 ^b^	17.13 ± 0.57 ^a^
Mean	71.70 ± 0.77	76.32 ± 1.36	80.36 ± 1.24	8.66 ± 0.76	17.67 ± 0.61

* *T_o_*: onset gelatinization temperature, *Tp:* peak gelatinization temperature, *Tc:* end gelatinization temperature, Δ*H* enthalpy of gelatinization. ** Mean values of varieties for each species in the same column are different significantly.

## Data Availability

The data presented in this study are available on request from the corresponding author. The data are not publicly available due to ongoing research using some of the data.
